# 1000 highly cited articles in depression research (2016–2026): A bibliometric and scoping review (global depression research: 2016–2026)

**DOI:** 10.1097/MD.0000000000050921

**Published:** 2026-09-25

**Authors:** Jinlei Li, Fen Ai, Yu Li, Bingxin Cheng, Zhen Chen

**Affiliations:** a Department of Emergency Medicine, The Central Hospital of Wuhan, Tongji Medical College, Huazhong University of Science and Technology, Wuhan, China; b School of Medicine, Jianghan University, Wuhan, China.

**Keywords:** bibliometrics, depression, major depressive disorder, scoping review, visual analysis

## Abstract

**Background:**

Depression is one of the world’s leading causes of disability, creating significant challenges for individuals and society. Although research in this field has grown rapidly, we still lack a complete understanding of how depression research has evolved globally over the past decade, especially regarding the long-term effects of the Coronavirus disease 2019pandemic.

**Methods:**

We collected data from the Web of Science Core Collection as of April 28, 2026. After careful screening, we included 1000 highly cited articles. Using bibliometric methods and visualization tools, we analyzed collaboration networks, keyword patterns, emerging topics, and timeline developments.

**Results:**

Our analysis shows that the United States produces more than half of the world’s high-impact depression research. Research focus has shifted from traditional theories to new areas like brain-immune connections and gut bacteria, leading to comprehensive treatment approaches that combine digital technology. The Coronavirus disease 2019 pandemic served as a major turning point, significantly reshaping research priorities and accelerating the integration of medicine and data science.

**Conclusions:**

Over the past decade, depression research has dynamically evolved in response to external challenges. Exploring basic biological mechanisms and combining knowledge from different fields have become central trends. Our comprehensive analysis provides valuable insights for planning future research, developing policies, and understanding the direction toward “multimodal integration” in depression treatment.

## 1. Introduction

Depression, also known as major depressive disorder, is a disabling mental illness characterized by persistent low mood, loss of interest, and diminished pleasure.^[1]^ As one of the most common mental health problems in the world, depression not only seriously affects the mental health of patients but also causes significant physical functional impairment and social dysfunction.^[2,3]^According to 2025 data from the World Health Organization, more than 1 billion people around the world are suffering from mental disorders, among which anxiety disorders and depression are the most common. These 2 types of diseases alone cause up to 1 trillion US dollars in global economic losses every year.

Bibliometrics is a research method that uses mathematical and statistical techniques to analyze scientific literature. In today’s data-rich environment, bibliometrics helps us objectively measure and understand how research fields develop and influence each other by examining authors, keywords, citations, and other factors.

In terms of biomedical research, scholars not only focus on traditional biomarkers,^[4]^ microglia-related mechanisms,^[5]^ and the comorbid relationship between depression and type 2 diabetes,^[6]^ but also deeply explore the relationship between intestinal microbiota and depression,^[7]^ as well as the specific pathological process of post stroke depression.^[8]^ At the same time, non-drug intervention and physical therapy have also attracted much attention, such as research on the intervention effects of Tai Chi training of different intensities on the “trinity” of fatigue, insomnia and depression in cancer patients,^[9]^ and the long-term application and development of electroencephalography technology in depression research.^[10]^ What is particularly significant is that artificial intelligence technology is becoming an important driving force in promoting depression research. Multiple studies have shown that data-driven methods have great potential in depression analysis,^[11]^ analysis of the global research landscape^[12]^ and comprehensive applications of insomnia, anxiety, and depression,^[13]^ marking that the field is moving towards a new stage of intelligence and precision.

Although Wang et al^[14]^analyzed depression research trends from 2004 to 2019, as of April 2026, no up-to-date systematic bibliometric analysis exists for this field globally.

To fill this gap, our study uses bibliometrics and scientific mapping methods to conduct a comprehensive analysis of 1000 highly cited depression articles from 2016 to 2026. We systematically examine global research output distribution, how research topics have evolved, patterns of collaboration between different fields, and how knowledge spreads over time.

To address this gap, we use a mixed-methods approach that combines bibliometric analysis (which tracks research trends and knowledge development) with scoping review techniques (which systematically examine the scope, volume, and key themes in existing literature). Based on this approach, our study has 3 clear goals:

Goal 1: Describe global depression research output patterns and how collaboration networks have evolved over time.

Goal 2: Understand how depression research topics have changed and how knowledge has developed.

Goal 3: Evaluate the impact of major external events, particularly the Coronavirus disease 2019 pandemic.

## 2. Methods

### 2.1. Literature sources and search strategy

We conducted a thorough search of the Web of Science (WoS) Core Collection to find relevant articles. We chose the Web of Science database as our primary source because it covers over 12,000 peer-reviewed journals and has proven reliability for bibliometric studies compared to other databases.^[15]^

On April 28, 2026, we used topic search methods with medical subject headings related to depression. Our specific search strategy is shown in Table 1.

**Table 1:** Search strategy for web of science core collection.

Database	Search strategy
Web of science	#1 TS=(“depression”) #2 TS=(“depressive symptom*” OR “symptom, depressive”) #3 TS=(“emotional depression” OR “depression, emotional”) #4 #1 OR #2 OR #3

Our initial search found 536,105 articles. We then applied strict inclusion and exclusion criteria through multiple screening rounds: first, we removed articles published before 2016 or after 2026 (n = 212,463); next, we excluded non-research documents such as conference abstracts, news reports, and brief communications (n = 31,860), as well as non-English articles (n = 3592). To ensure topic relevance, we also removed articles that used the word “depression” in non-mental health contexts (such as economic downturns or suppression of functions, n = 287,190). Finally, we kept 1000 highly cited articles for our bibliometric analysis (Figure 1 and Supplementary Table S1, Supplemental Digital Content 1).

**Figure 1.**
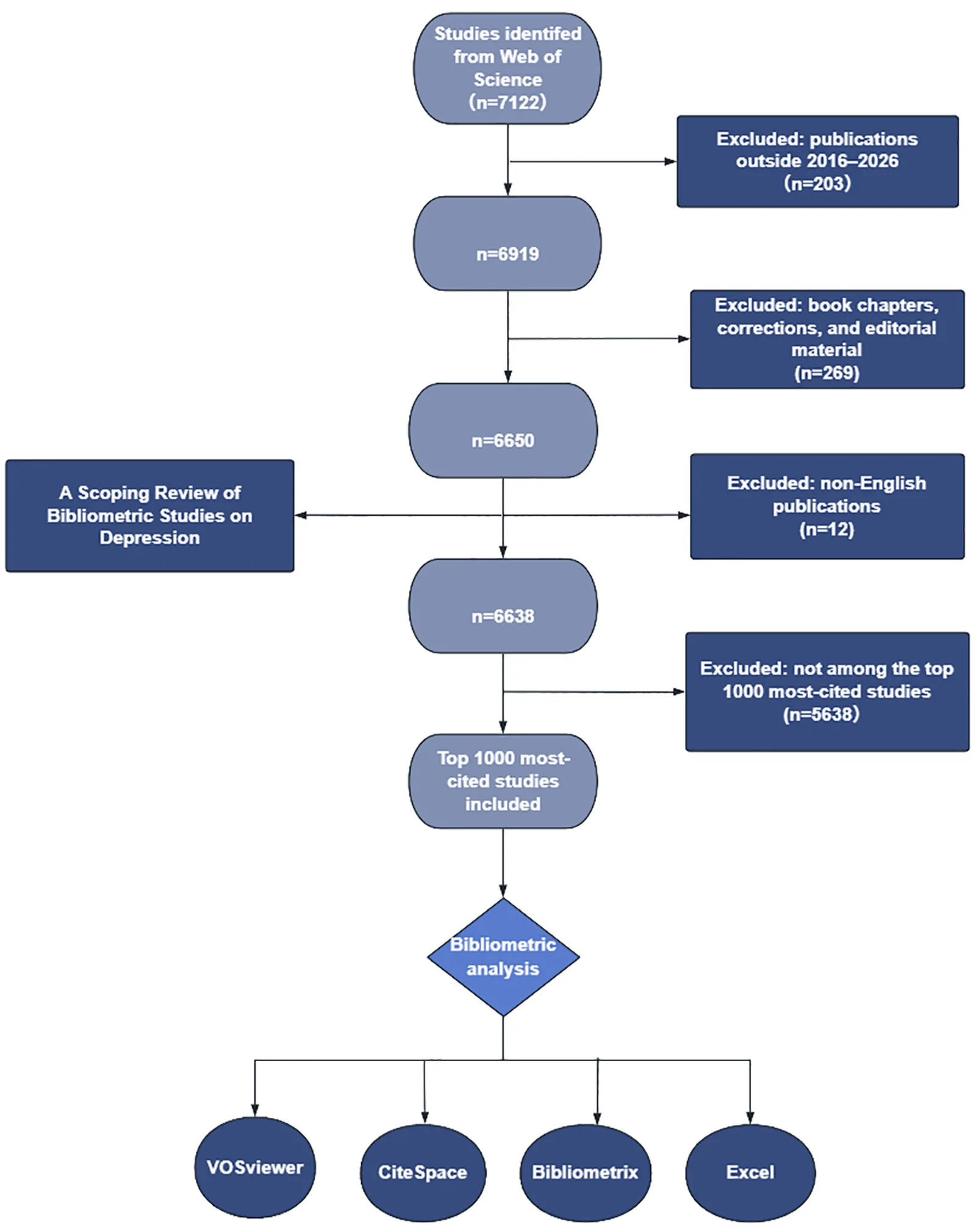
PRISMA flow diagram of the literature screening and selection process for the 1000 highly cited depression articles.

### 2.2. Eligibility criteria and screening process

Literature screening was conducted strictly following the preferred reporting items for systematic reviews and Meta-Analyses extension for Scoping Reviews guidelines. The inclusion criteria are clearly defined as: The article content is directly related to depression research; Literature published in English; Article types are limited to original research and review articles;and Included in the SCI Core Collection.

### 2.3. Data collection and statistics

A mixed-methods approach was employed, integrating bibliometric analysis with scoping review methodology. The data were analyzed using the following software:

VOSviewer (Version 1.6.20)^[16]^: Used for constructing and visualizing bibliometric networks, including co-authorship networks (countries, institutions, authors), keyword co-occurrence analysis, and journal coupling. This tool helped map the conceptual structure and collaboration patterns.^[17]^

Bibliometrix (R Package; Version 4.5.0)^[18]^: Employed to generate heatmaps visualizing the temporal distribution of keywords and to perform descriptive statistics, providing insights into research topic evolution over time.^[19]^

CiteSpace (Version 6.3.R1)^[20,21]^: Utilized for detecting knowledge domain bursts and visualizing dual-map overlays to explore the intellectual foundation and emerging trends within the literature.

Scimago Graphica (Version 1.0.0.2)^[22]^: Specifically employed to visualize geographic distribution and research output across countries on a global map.

Microsoft Excel: Used for analyzing monthly publication trends from 2016 to 2026.

### 2.4. Project registration and ethical considerations

This scoping review has been prospectively registered on the Open science framework platform.^[23]^As this study is based on secondary data from existing publications and does not involve direct interaction with human participants or animals, ethical approval was not required.

## 3. Results

### 3.1. Analysis of publication trends and basic quantitative characteristics

We used Bibliometrix software to systematically analyze 1000 highly cited depression articles from 2016 to 2024 (Fig. 2). Figure 3 shows how research patterns have changed over time. Although our study plan covered 2016-2026, the actual analysis ended in 2024. This isn’t a design flaw but follows standard bibliometric practice - it takes 2 to 3 years for articles to accumulate enough citations to show stable patterns, and 2025–2026 data isn’t yet mature enough for analysis.

**Figure 2.**
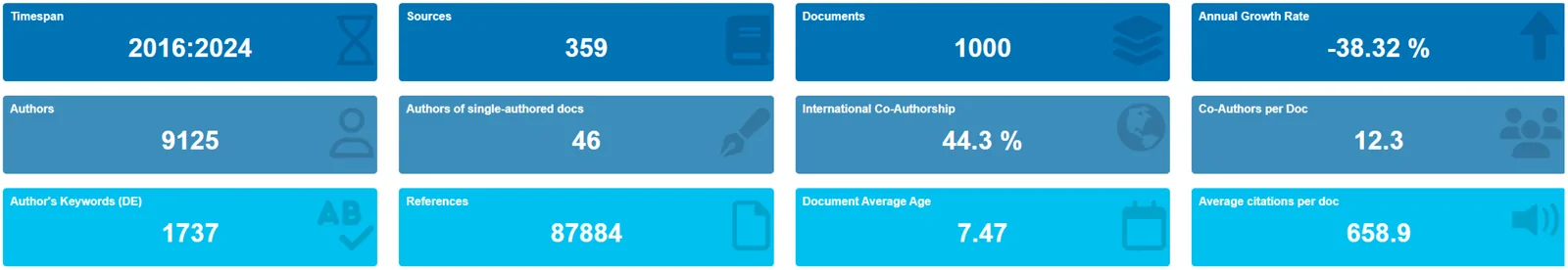
Publication trends and basic quantitative characteristics of the 1000 highly cited depression articles from 2016 to 2024.

**Figure 3.**
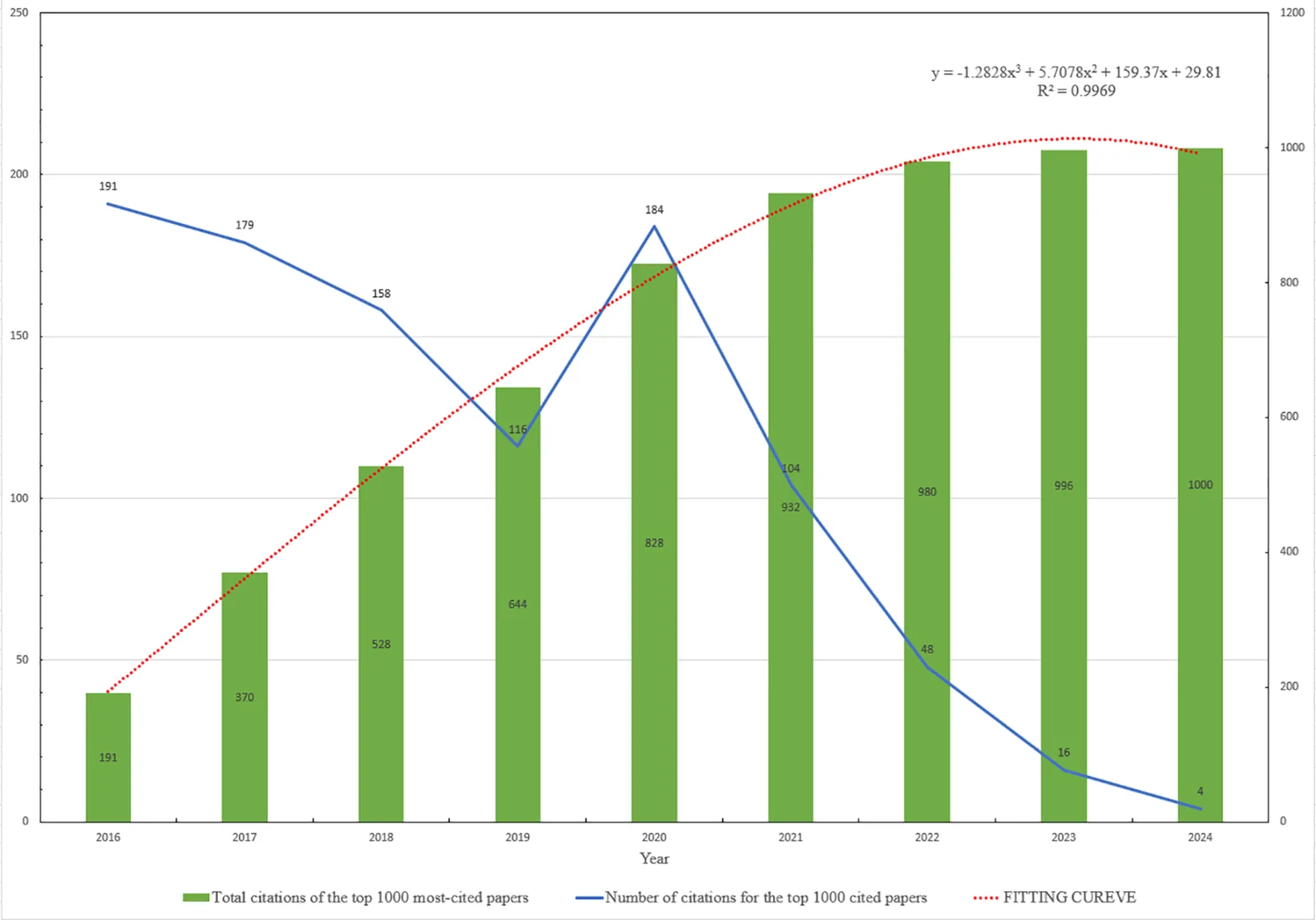
Temporal evolution of research patterns in highly cited depression articles from 2016 to 2024.

### 3.2. Author-author collaboration network analysis

Our study identifies key contributors to depression research. As shown in Table 2, the top ten authors published 102 papers (10.2% of all articles). Carvalho, Andre F., Stubbs, Brendon, and Firth, Joseph tied for first place, each publishing 12 papers, showing their consistent contributions to this field.

**Table 2:** Ranking of highly productive authors and cited authors (Top 10).

Rank	Author	Documents	Rank	Co-cited author	Documents
1	Carvalho, Andre F.	12	1	Kessler, Rc	236
2	Stubbs, Brendon	12	2	Culipers, P	152
3	Firth, Joseph	12	3	Kroenke, K	130
4	Cuijpers, Pim	11	4	Moher, D	123
5	Patel, Vikram	11	5	Higgins, Jpt	116
6	Mcintyre, Roger S.	10	6	Carhart-Harris, Rl	105
7	Berk, Michael	9	7	Beck, At	104
8	Solimi, Marco	9	8	World Health, Organization	103
9	Sanacora, Gerard	8	9	Maes, M	96
10	Vancampfort, Davy	8	10	Kendler, Ks	90

In citation analysis, Kessler, RC ranked highest. His work on depression epidemiology and diagnostic criteria has laid important groundwork for the field. Culipers, P’s research on evidence-based psychotherapy and Kroenke, K’s development of depression assessment tools have also been widely recognized. Notably, Carhart-Harris, RL’s pioneering work on psychedelic treatments for depression has entered the top ten citations, reflecting growing interest in innovative treatments.

Figure 4A shows several research teams centered around scholars like Carvalho, McIntyre, Cuijpers, and Manji. Figure 4B divides the author network into 6 major research groups:

**Figure 4.**
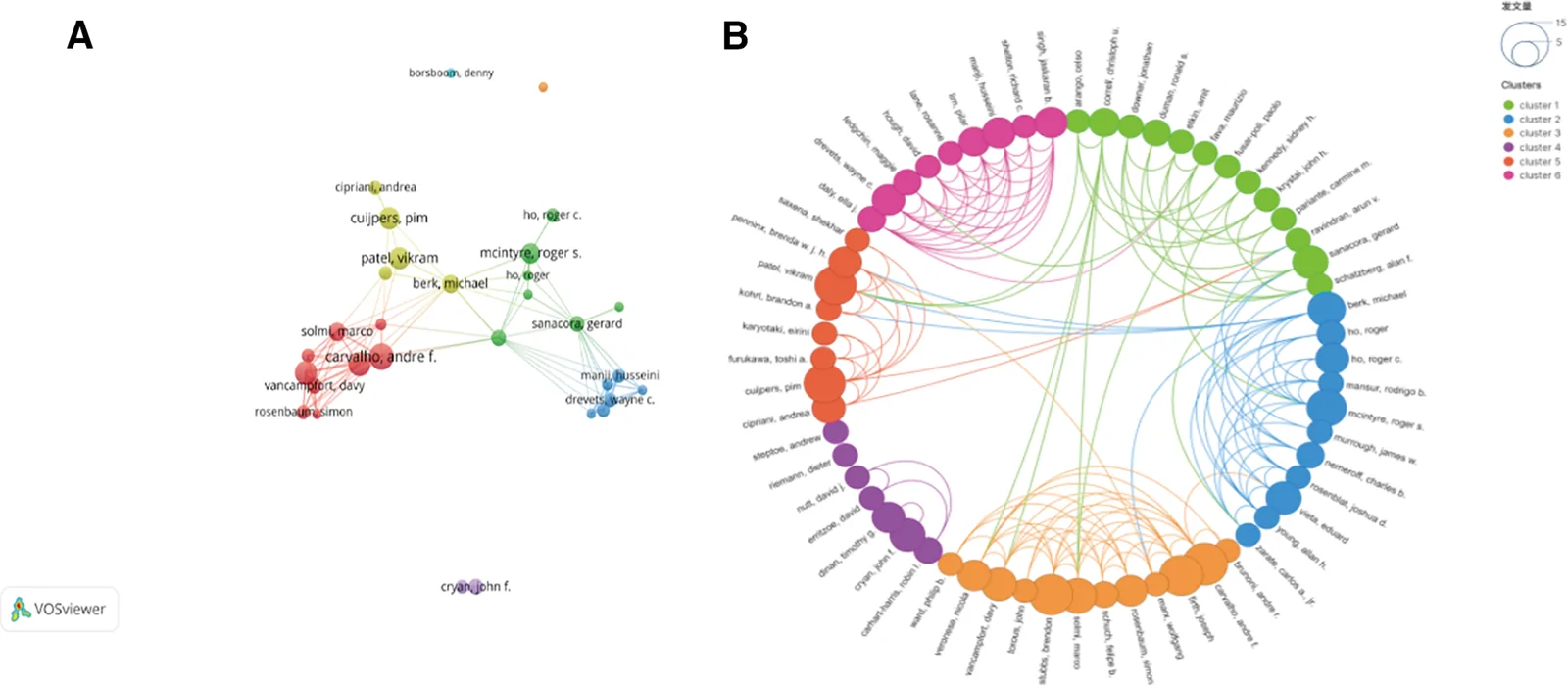
(A) Author collaboration network visual analysis; (B) Author collaboration cluster analysis, identifying 6 major research groups.

Cluster 1 (led by Carvalho and Stubbs): Focuses on antidepressant drug effectiveness.

Cluster 2 (led by Cuijpers): Focuses on evidence-based psychotherapy research.

Cluster 3 (led by Patel) focuses on global mental health service accessibility.

Clusters 4, 5, and 6 focus on biological markers, brain stimulation technology, and public health intervention strategies, respectively.

### 3.3. Analysis of countries and national cooperation networks

The 1000 papers come from 81 countries. Table 3 shows the measurement characteristics of the top 10 countries. The United States ranked first, with 533 articles published, accounting for 53.3% of the total. The United Kingdom and Canada ranked second and third, with 261 and 148 articles, respectively. It is worth noting that China’s average citation frequency for a single article reached 796.23 times, ranking first among the top ten countries.

**Table 3:** Top ten countries’ metrological characteristics.

RANK	Country	Documents	Citations	Percentage (%)	Citation per publication	Total link strength
1	USA	533	340,023	53.3%	637.94	1092
2	ENGLAND	261	184,434	26.1%	706.64	932
3	CANADA	148	108,490	14.8%	733.04	644
4	AUSTRALIA	144	98,580	14.4%	684.58	696
5	CHINA	113	89,974	11.3%	796.23	353
6	GERMANY	106	75,627	10.6%	713.46	629
7	NETHERLANDS	101	68,607	10.1%	679.28	533
8	ITALY	89	64,313	8.9%	722.62	508
9	SPAIN	72	51,418	7.2%	714.14	496
10	SWEDEN	52	40,504	5.2%	778.92	352

Figure 5 comprehensively reveals the geographical distribution pattern and cooperation network of global depression research in the past ten years through 4 sets of complementary visualization charts: The bubble chart intuitively shows the significant advantages in the number of publications and influence of core countries such as the United States, the United Kingdom, and China. The world map network diagram clearly depicts geographical connections. It draws a close international cooperation network with the United States and Britain as dual hubs, radiating to Canada, Australia, and European countries. The ring diagram and the radial diagram deeply analyze the flow and hierarchical structure of cooperation, highlighting the leading role of “U.S.-UK-Canada-Australia” in global knowledge flows and its radiation effect on peripheral countries.

**Figure 5.**
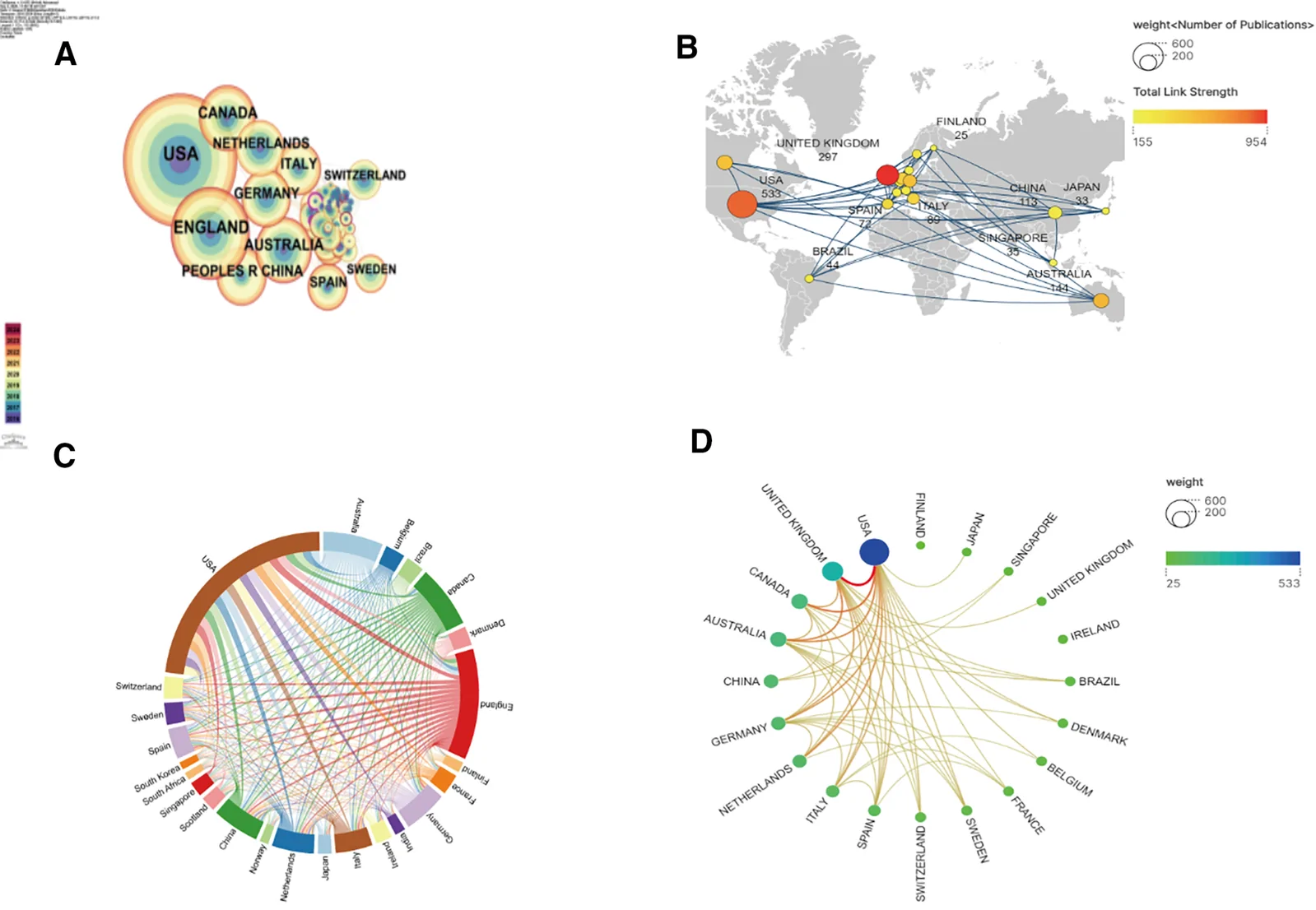
Geographical distribution and international cooperation network of global depression research over the past decade. (A) Bubble chart; (B) world map network; (C) ring diagram; (D) radial diagram.

### 3.4. Analysis of institutions and institutional cooperation networks

Our study included 1858 research institutions worldwide. The top ten institutions alone published 435 papers (43.5% of the total), showing how concentrated depression research resources are (Table 4). The United States has the strongest institutional presence, with 5 of the top 10 institutions being American (Harvard Medical School, Stanford University, Massachusetts General Hospital, Columbia University, and University of Pittsburgh).

**Table 4:** Metric characteristics of the top ten institutions by publication volume.

Rank	Organization	Country	Documents	Citations	Average citation
1	Kings Coll London	United Kingdom	69	54,523	790.19
2	Harvard Med Sch	USA	65	39,433	606.66
3	Univ Toronto	Canada	44	34,255	778.52
4	Univ Oxford	United Kingdom	43	26,861	624.67
5	Ucl	United Kingdom	41	30,490	743.66
6	Stanford Univ	USA	40	26,405	660.12
7	Univ Melbourne	Australia	33	20,916	633.82
8	Massachusetts Gen Hosp	USA	31	19,225	620.16
9	Columbia Univ	USA	30	23,374	779.13
10	Univ Pittsburgh	USA	29	19,732	680.41

Figure 6A shows 3 major academic hubs: King’s College London, Harvard Medical School, and Oxford University. Figure 6B divides institutions into 6 research clusters:

**Figure 6.**
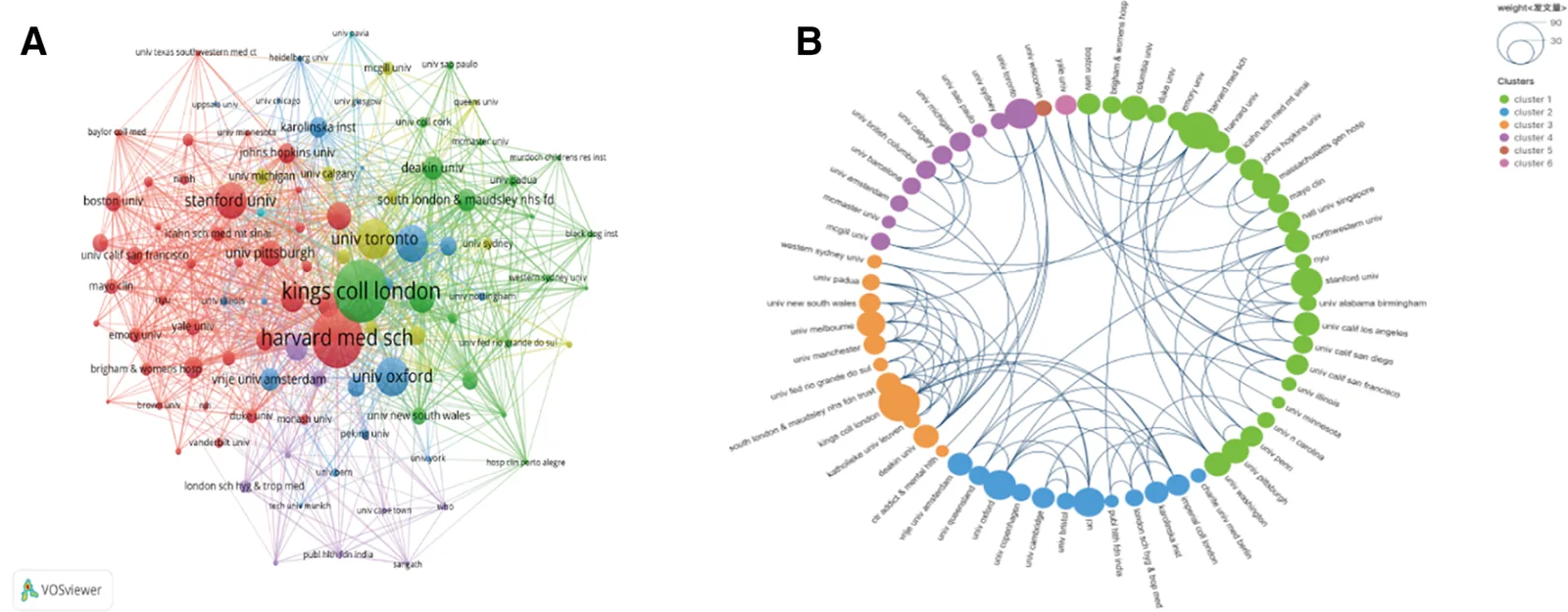
(A) Institutional cooperation network; (B) Institutional cluster distribution, dividing institutions into 6 research clusters.

Cluster 1 (blue): Led by King’s College London, focuses on depression epidemiology and public health interventions.

Cluster 2 (red): Led by Harvard Medical School, studies brain mechanisms and biological markers of depression.

Cluster 3 (green): Centered at the University of Toronto, specializes in clinical treatment and effectiveness evaluation.

Cluster 4 (yellow): Researches genetic and molecular foundations of depression.

Cluster 5 (purple): Explores cognitive neuroscience aspects of depression.

Cluster 6 (orange): Studies social and psychological factors in depression prevention.

### 3.5. Analysis of journal publication volume and co-citations

The 1000 papers were published across 359 different academic journals. The top 5 journals are all leading publications in psychiatry and clinical medicine, with *The Lancet* ranking first (36 articles). The top 10 journals published 289 papers total (28.9% of all articles), as shown in Figure 7.

**Figure 7.**
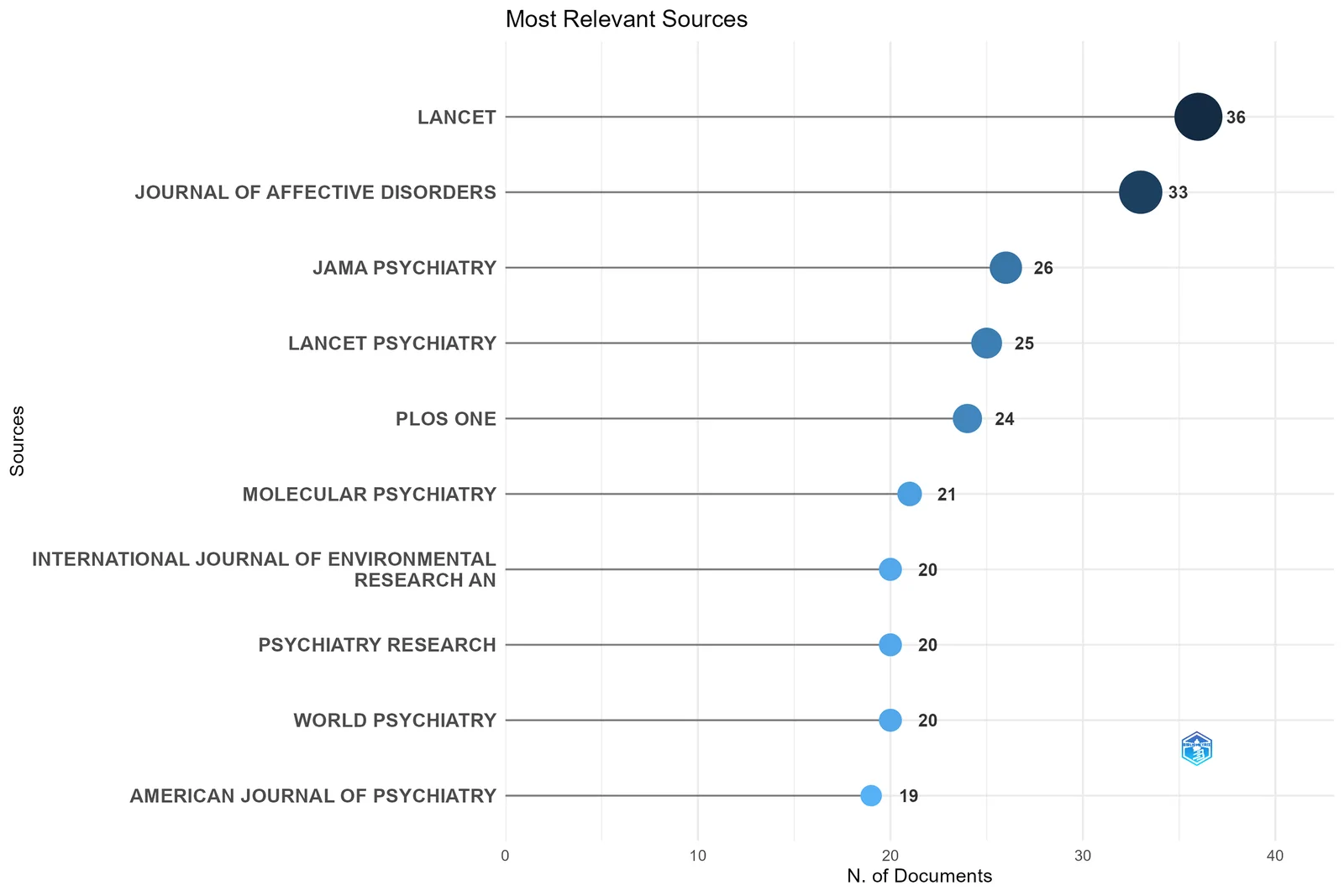
Distribution of major publishing journals (Top 10) in depression research.

When we analyzed which journals were most frequently cited together, *Journal of Affective Disorders* emerged as the field’s core knowledge source with 1776 co-citations (Table 5). Importantly, journal citations show a strong concentration pattern the top 10 journals account for most citations in the field, indicating that depression research findings are heavily concentrated in a few high-impact journals (Fig. 8).

**Table 5:** Journal citation analysis (Top 10).

Rank	Cited Journal	Co-Citation	IF (2025)	Quartile in category
1	J AFFECT DISORDERS	1776	4.9	Q1
2	BIOL PSYCHIAT	1485	9	Q1
3	PLOS 1	1468	2.6	Q2
4	AM J PSYCHIAT	1373	14.7	Q1
5	J NEUROSCI	1317	4	Q1
6	LANCET	1274	88.5	Q1
7	P NATL ACAD SCI USA	1027	9.1	Q1
8	ARCH GEN PSYCHIAT	1023	–	–
9	JAMA-J AM MED ASSOC	963	55	Q1
10	NATURE	943	48.5	Q1

**Figure 8.**
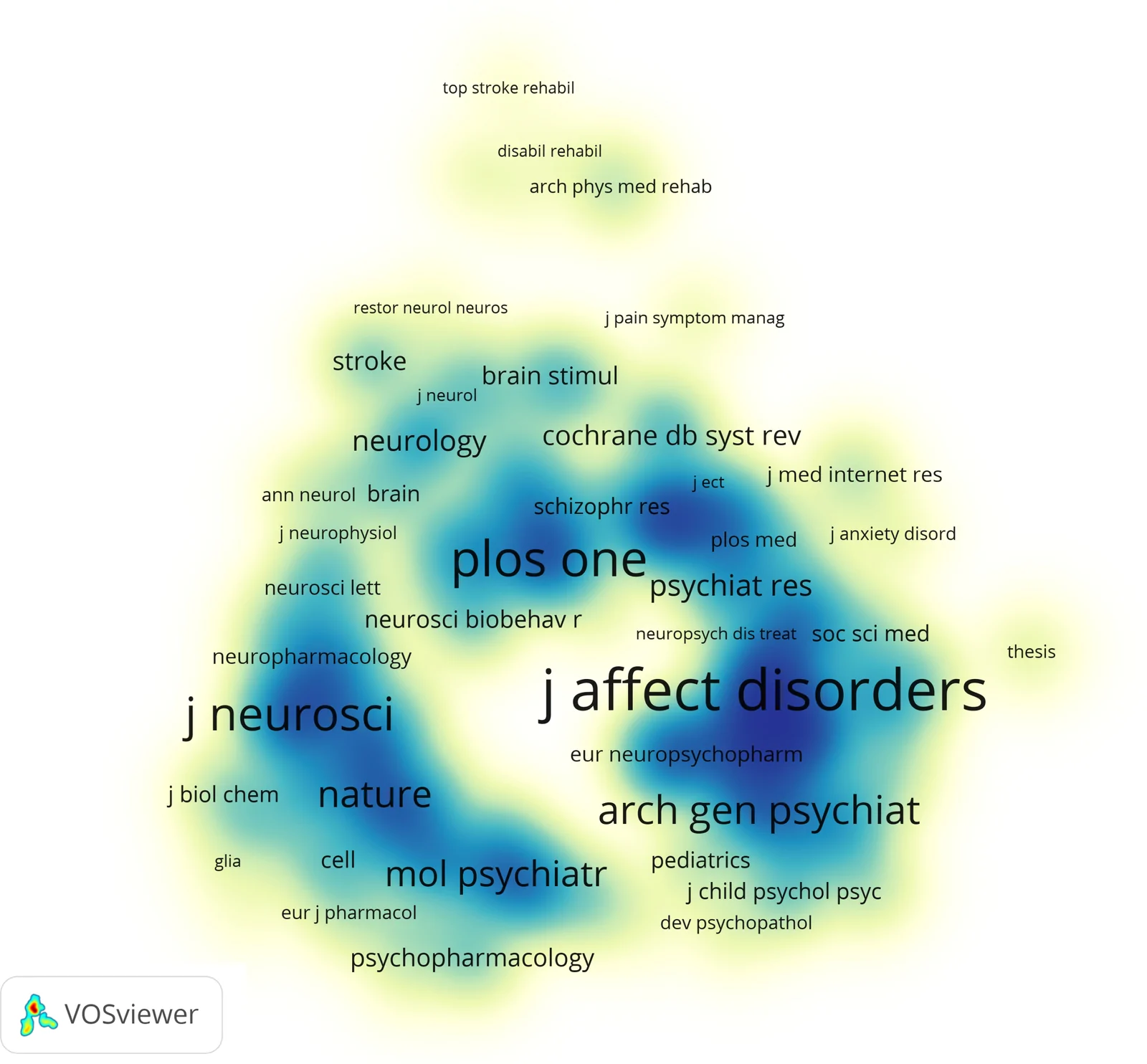
Heat map of the distribution of highly cited literature journals.

### 3.6. Topic distribution

Figure 9 shows how depression research has evolved over the past decade from studying single causes to becoming a highly interconnected, multidisciplinary knowledge system. Research now spans the full spectrum from basic brain mechanisms to clinical treatments, from individual care to public health strategies.

**Figure 9.**
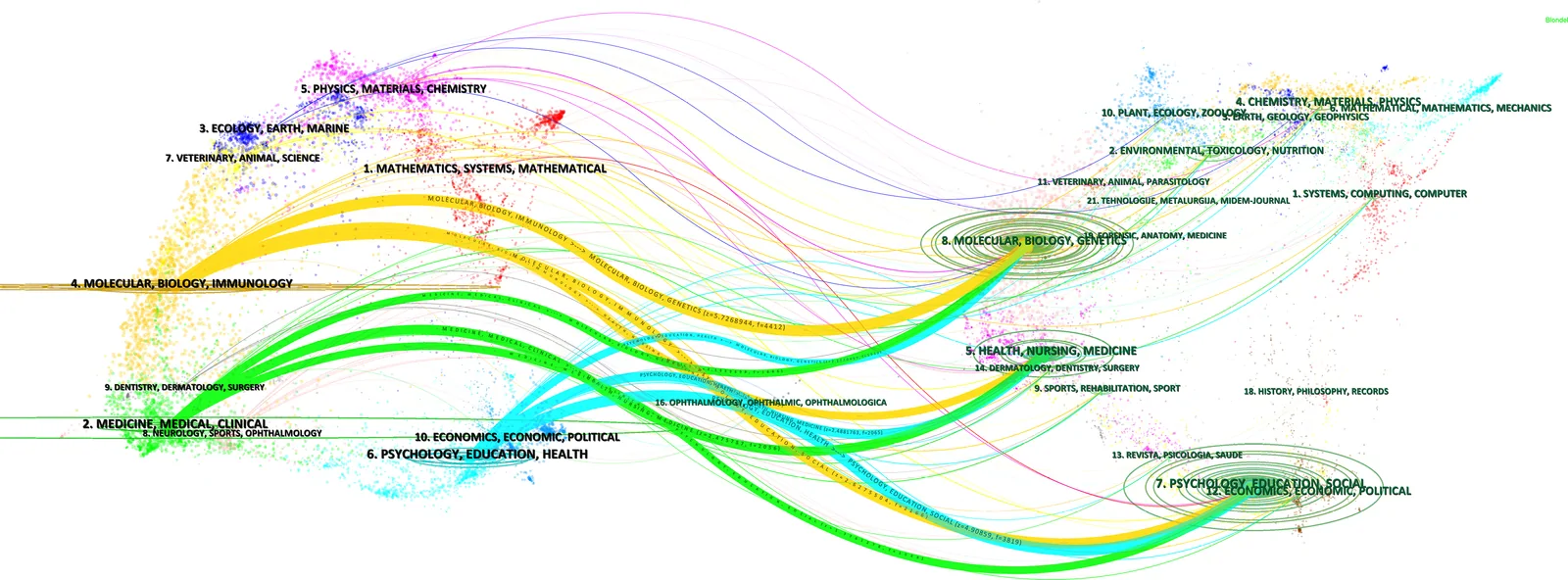
Knowledge flow network visualization diagram showing the evolution of depression research over the past decade.

### 3.7. Keyword analysis

Table 6 shows that “Depression” (441 times) and “Major Depression” (141 times) are used as core keywords; secondly, comorbidities and related disorders (such as Anxiety, Bipolar Disorder, Schizophrenia) and specific groups (Adolescents, Children, Adults) are frequently paid attention to, indicating that research focuses on clinical complexity and population heterogeneity. The public health perspective (Mental Health, 191 times), epidemiological characteristics (Prevalence, 85 times), and environmental factors (Covid-19, 95 times) highlight the profound social macro-background that shapes depression research. At the same time, both intervention strategies and physiological mechanisms are emphasized, reflecting “psycho-biological” integration. Keywords such as adverse clinical outcomes, patient functional impairment, and physical comorbidities indicate that research is not only focused on the disease itself but also on its broader impact on the overall health of the individual.

**Table 6:** Statistical analysis of high-frequency keywords (Top20).

Rank	Keyword	Occurrences	Core domain	Rank	Keyword	Occurrences	Core domain
1	Depression	441	Core disorder	11	Treatment-resistant depression	31	Clinical phenotype
2	Mental health	191	Public health	12	Schizophrenia	31	Related disorders
3	Anxiety	179	Comorbid symptoms	13	Adolescents	30	Study population
4	Major depression	141	Core disorder	14	Children	30	Study population
5	Covid-19	95	Environmental context	15	Adults	27	Study population
6	Stress	94	Psychological mechanisms	16	Suicide	24	Clinical outcome
7	Prevalence	85	Epidemiology	17	Sleep	23	Physiological mechanisms
8	Quality-Of-Life	83	Patient outcomes	18	Inflammation	22	Biological mechanisms
9	Cognitive-behavioral therapy	53	Intervention strategy	19	Physical activity	21	Lifestyle factors
10	Bipolar disorder	32	Related disorders	20	Obesity	20	Somatic comorbidities

Figure 10A clearly presents the network structure with “depression,” “mental health” and “anxiety” as the core. In Figure 10B, the core clusters include traditional research directions such as mental health maintenance and postpartum depression. At the same time, clusters such as “double blind” and “validity” reflect the rigorous requirements of clinical research methodology. Of particular note is “irritable bowel” The emergence of the “syndrome” cluster reveals the interdisciplinary penetration of the brain-gut axis mechanism in the pathophysiology of depression. In Figure 10C, the research focus is undergoing a transfer process from early basic pathological descriptions (2016–2018) to specific interventions such as cognitive behavioral therapy (2019–2021) and longitudinal follow-up studies of specific populations (2022–2024), while “inflammation” and “gut Topics such as” “microbiota” appear throughout the entire research period; In Figure 10D, “outbreak” showed the strongest emergence intensity in 2020, “psychological impact” had an emergence intensity of 9.76 in 2020, and “anxiety” has continued to emerge since 2021, with an intensity of 4.99. These emergent words completely record research hot spots covering psychopathology, clinical trials, social psychological factors, physiological indicators, and other fields from the temporal dimension.

**Figure 10.**
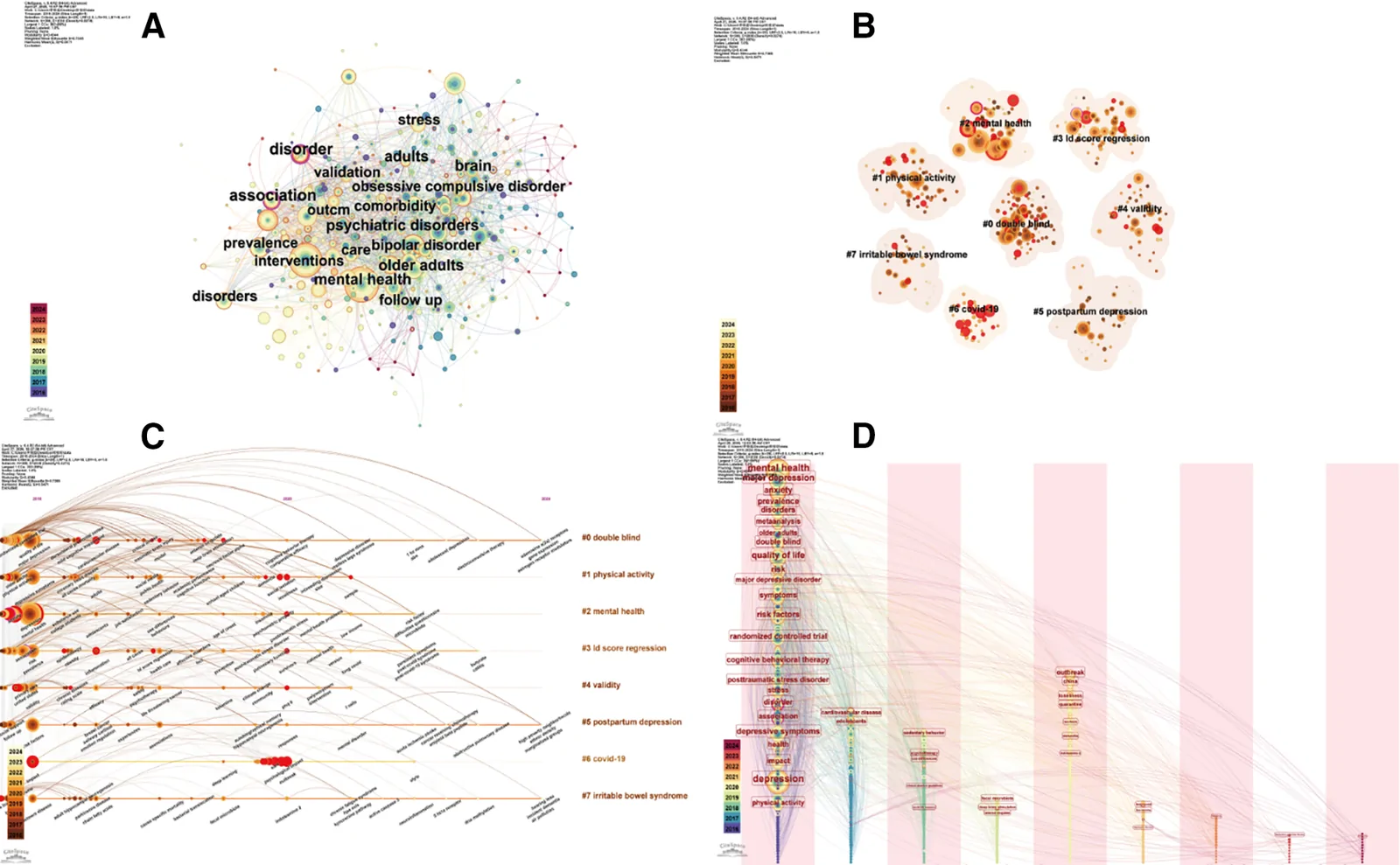
(A) Keyword co-occurrence network; (B) Keyword cluster analysis; (C) Timeline view of keyword evolution; (D) Burst detection of keywords in depression research from 2016 to 2024.

From 2016 to 2019, research was dominated by basic research on psychopathology and clinical trial methodology. From 2020 to 2022, the COVID-19 epidemic became a watershed in the research agenda, with keywords such as “COVID-19,” “pandemic,” and “quarantine” appearing explosively. From 2023 to 2024, the emergence intensity of keywords such as “gut-brain axis,” “neuroinflammation,” and “digital intervention” will increase significantly. At the same time, the continued activity of “physical activity” and “lifestyle factors” indicates that non-pharmacological intervention strategies have received increasing attention from the academic community (Fig. 11).

**Figure 11.**
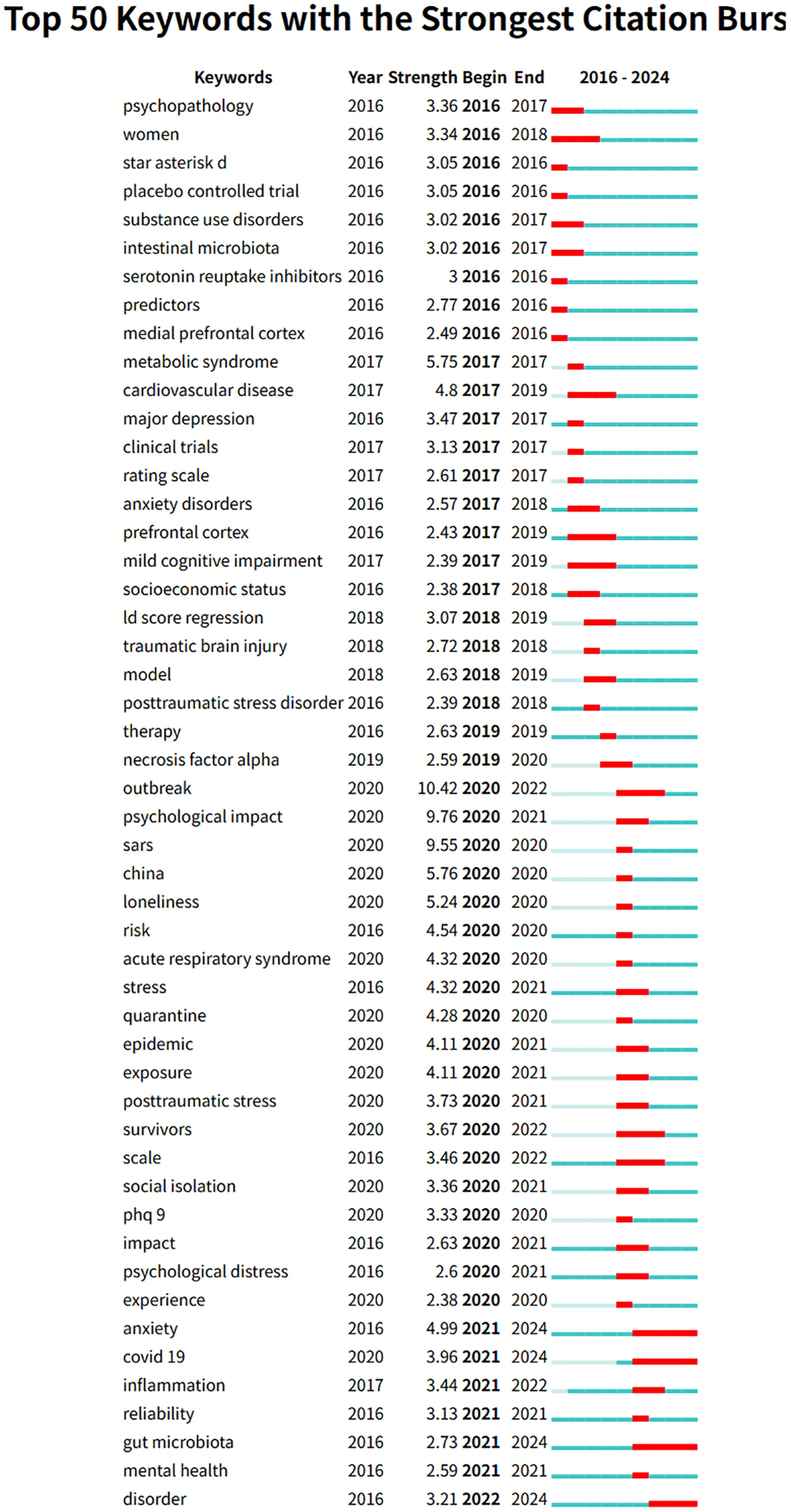
Visualization of keyword emergence (citation burst) intensity and time distribution (Top 50 keywords with the strongest citation bursts, 2016–2024).

### 3.8. Core literature and cluster analysis

Table 7 shows the distribution characteristics of the ten most frequently cited core documents in the field of depression research. Research related to the COVID-19 epidemic occupies a prominent position in the highly cited literature. The top four are all studies on the mental health impact of the epidemic published in 2020.^[24–33]^ Among them, “The psychological impact of quarantine and how to reduce it: rapid review of the evidence” by Brooks et al ranks first with 6965 citations.

**Table 7:** The ten most cited core documents.

Rank	Article title	Source	Authors	Yr	Number of citations
1	“The psychological impact of quarantine and how to reduce it: rapid review of the evidence”	Lancet	Brooks et al	2020	6965
2	“Immediate psychological responses and associated factors during the initial stage of the 2019 Coronavirus disease (COVID-19) Epidemic among the General Population in China”	International Journal Of Environmental Research And Public Health	Wang et al	2020	5180
3	“Factors associated with mental health outcomes among health care workers exposed to Coronavirus Disease 2019”	Jama network open	Lai et al	2020	4846
4	“Impact of COVID-19 pandemic on mental health in the general population: A systematic review”	Journal Of affective disorders	Xiong et al	2020	3636
5	“Depression”	Lancet	Malhi et al	2018	3285
6	“The role of inflammation in depression: from evolutionary imperative to modern treatment target”	Nature reviews immunology	Miller et al	2016	2939
7	“Global, regional, and national incidence, prevalence, and years lived with disability for 310 diseases and injuries, 1990–2015: a systematic analysis for the global burden of disease study 2015”	Lancet	Vos et al	2016	2745
8	“Inflammageing: chronic inflammation in aging, cardiovascular disease, and frailty”	Nature reviews Cardiology	Ferrucci et al	2018	2737
9	“Age at onset of mental disorders worldwide: large-scale meta-analysis of 192 epidemiological studies”	Molecular psychiatry	Solmi et al	2021	2694
10	“The Fear of COVID-19 Scale: development and initial validation”	International journal Of mental health And addiction	Ahorsu et al	2020	2657

In terms of research topics, highly cited documents cover multiple dimensions such as the psychological impact of the epidemic, inflammatory mechanisms, global disease burden, and epidemiological characteristics. Among them, Miller et al study on the role of inflammation in depression (2939 citations) and Malhi et al review of depression (3285 citations) constitute an important theoretical foundation in this field, while Solmi et al large-scale meta-analysis on the age of onset of mental disorders (2694 citations) provides key evidence for early clinical intervention.

The left side of Figure 12 clearly shows that since 2020, literature nodes represented by scholars such as Bao YP, Cao WJ, Xiang YT, and Brooks SK have emerged intensively. This phenomenon intuitively reflects that after the outbreak of the new crown epidemic, research on the impact of the epidemic on mental health quickly became the most active knowledge base in this field. The cluster view on the right side of the map divides the literature network into multiple topic groups, among which clusters such as “#7 COVID–19 pandemic,” “#13 COVID–19 syndrome” and “#4 healthcare worker” highlight the core focus of recent research; at the same time, clusters such as “#1 gut-brain axis,” “#9 gut microbiota” and “#12 treatment-resistant depression” reflect the continued in-depth exploration of pathological mechanisms and clinical treatment in this field.

**Figure 12.**
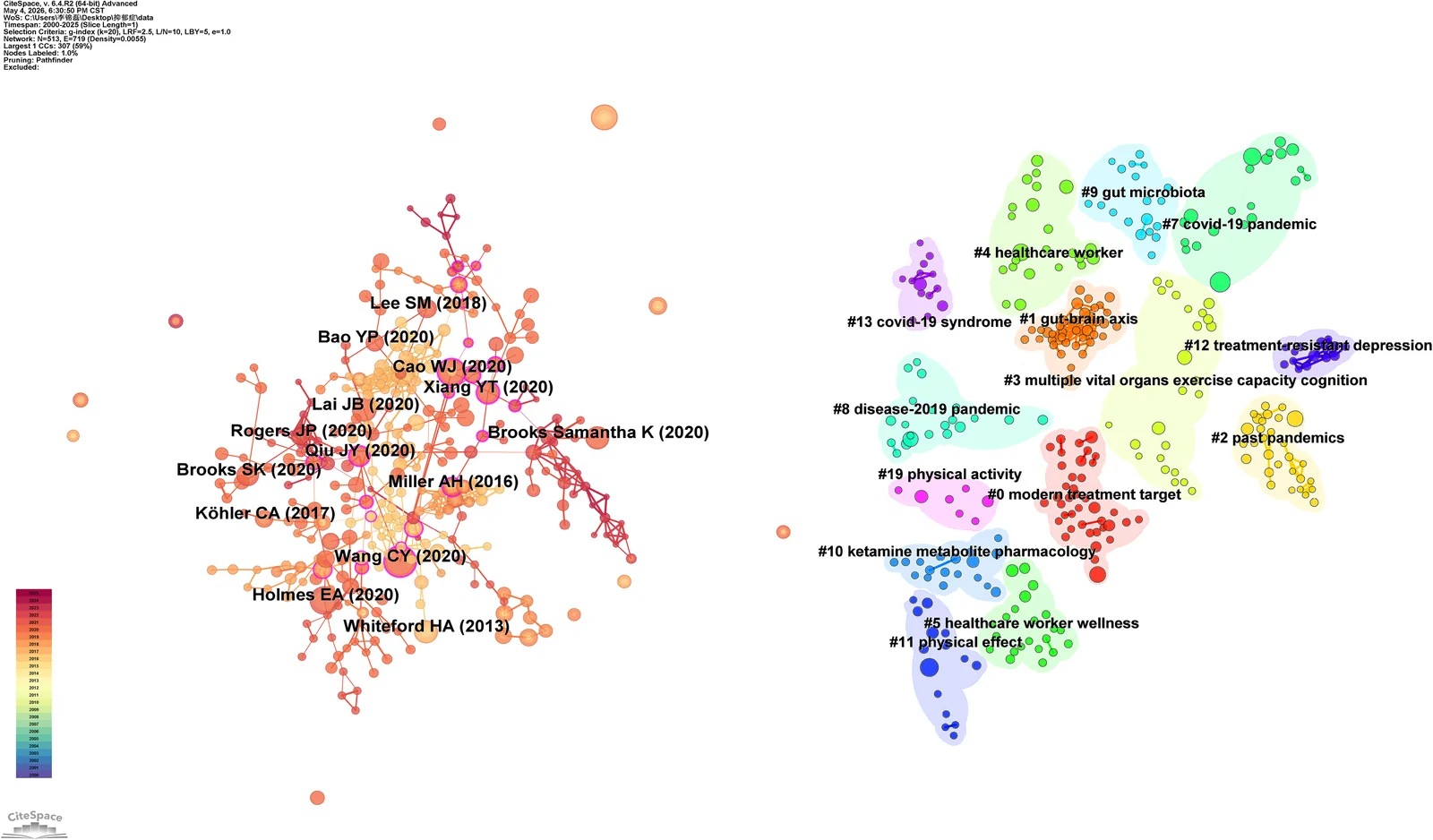
Document co-citation network and clustering, highlighting the impact of the COVID-19 pandemic and core research themes.

Figure 13 further reveals the evolutionary trajectory of topic clustering. Clusters such as “#0 modern treatment targets,” “#1 gut-brain axis” and “#9 gut microbiota” have a long time span (2016-2026). It is worth noting that since 2020, a large number of emerging clusters related to the epidemic have appeared in the graph, such as “#7 COVID–19 pandemic,” “#8 disease-2019 pandemic” and “#4 healthcare worker” for specific groups. These clusters have shown significant explosive growth between 2020 and 2022, intuitively reflecting the immediate role of the global public health crisis on the depression research agenda. In addition, clusters such as “#12 treatment-resistant depression” and “#10 ketamine metabolite pharmacology” reveal the continued exploration of refractory cases and new drug mechanisms in the clinical treatment field.

**Figure 13.**
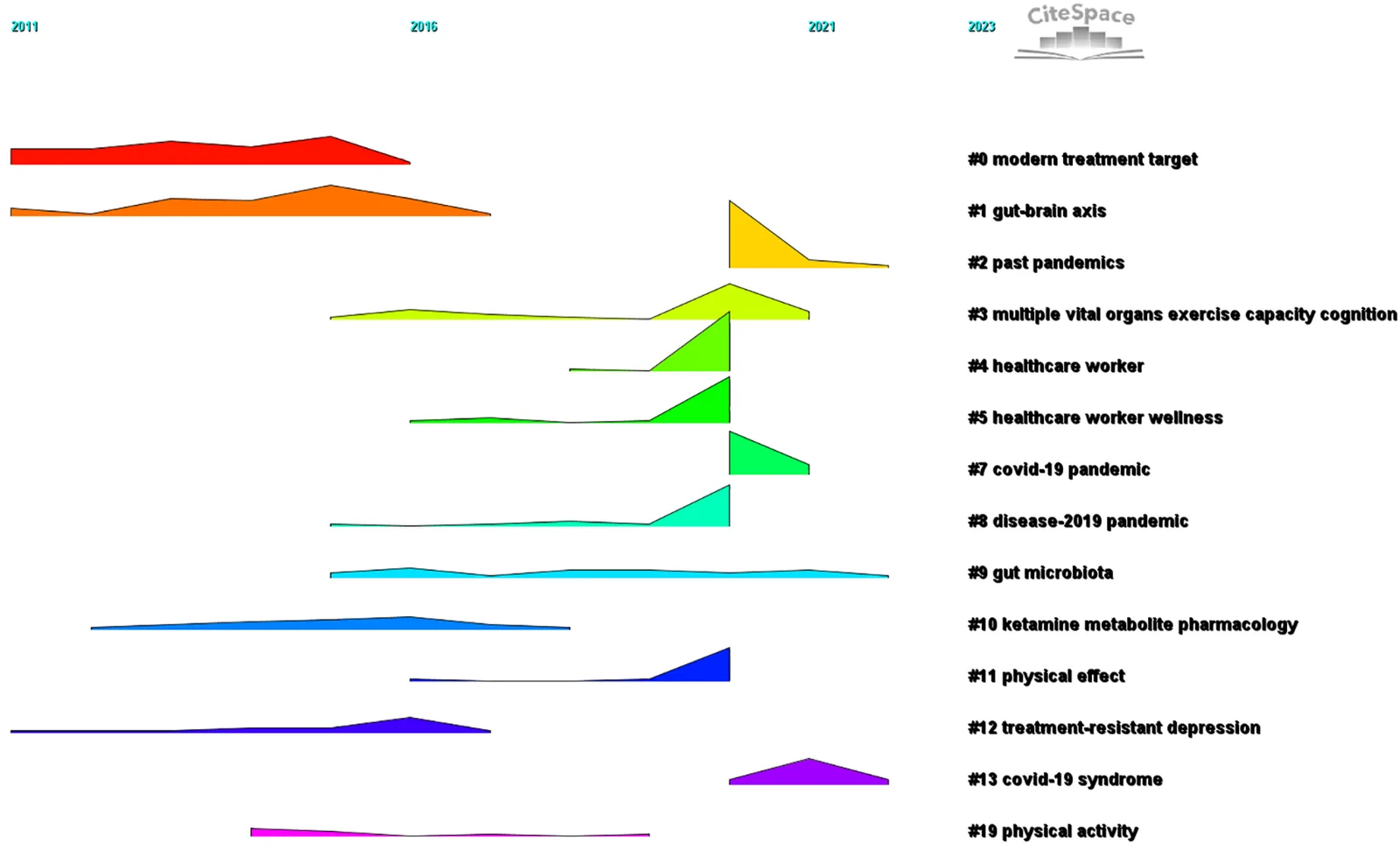
Evolution trajectory of topic clustering, revealing the temporal dynamics of research themes from 2016 to 2026.

### 3.9. A review of the scope of bibliometric research related to depression

From the bibliometric studies related to depression published between 2016 and 2026, a total of 479 initial documents were obtained through a systematic search integrating special bibliometric search terms. After strict screening to eliminate literature that strayed from the topic and did not focus on the core discussion of depression, 63 high-quality pieces of literature were finally included. The remaining documents were sorted according to their citation frequency, and the top 10 highly cited documents were selected for focused analysis (Table 8).^[14,34–42]^

**Table 8:** Top 10 highly cited bibliometric studies.

RANK	Article title	Authors (Yr)	Number of citations
1	Bibliometric and visual analysis of research on the links between the gut microbiota and depression From 1999 to 2019	Zhu et al(2021)	75
2	The current research landscape on the artificial intelligence application in the management of depressive disorders: A bibliometric analysis	Tran et al (2019)	54
3	Atrial fibrillation and depression: A bibliometric analysis from 2001 to 2021	Ai et al (2022)	42
4	A Bird’s-Eye view of exercise intervention in treating depression among teenagers in the Last 20 yr: A bibliometric study and visualization analysis	You et al (2021)	40
5	Bibliometric Study of the comorbidity of pain and depression research	Wang et al (2019)	33
6	Evolution and emerging trends in depression research from 2004 to 2019: A literature visualization analysis	Wang et al (2021)	31
7	Bibliometric mapping of the landscape and structure of nutrition and depression research: visualization analysis	Zyoud et al (2023)	28
8	A bibliometric analysis of research on heart failure comorbid with depression from 2002 to 2021	Ying et al (2023)	27
9	Parkinson’s disease related depression and anxiety: A 22-yr bibliometric analysis (2000–2022)	Zhang et al (2023)	27
10	Current global research landscape on COVID-19 and depressive disorders: bibliometric and visualization analysis	Al-Jabi et al (2021)	27

First, the focus of bibliometric research has evolved from descriptive statistics to mechanism-driven analysis. While early studies provided general overviews, recent high-impact analyses increasingly target specific biological pathways. Notably, Zhu et al work on the “gut microbiota-depression link” underscores the intense academic interest in the microbiome-gut-brain axis, marking a frontier in physiological research. Second, there is a growing emphasis on multimodal interventions and comorbidity management. Top-cited literature reveals a paradigm shift toward non-pharmacological strategies, including artificial intelligence driven management, exercise interventions for adolescents, and nutritional psychiatry. Furthermore, significant attention is directed at the bidirectional relationship between depression and somatic diseases. Highly cited works addressing comorbidities such as atrial fibrillation, heart failure, Parkinson’s disease, and pain indicate a clinical prioritization of the “depression-somatic” interaction. Finally, external global events have left an indelible mark on the research agenda. The impact of the COVID-19 pandemic is prominent, exemplified by Al-Jabi et al analysis of “COVID–19 and Depressive Disorders.” This confirms that social and environmental factors dynamically reshape research priorities, pushing the field toward an integrated model combining biomedical science with data-driven approaches.

## 4. Discussion

### 4.1. Comparative analysis with existing literature

Through a systematic review of existing bibliometric studies on depression, we found that the existing literature mainly presents 3 major limitations. First, the time span is limited. For example, 1 study only covers the period from 2004 to 2019 and fails to capture key research trends over the past 5 years. Although some studies involve recent research, they started late and have a limited topical scope. Second, the topic focus is narrow. Some studies concentrate on the top 100 highly cited papers on biomarkers; others are limited to microglia mechanisms; still others target specific comorbidities such as type 2 diabetes and post-stroke depression, lacking a panoramic analysis of the field. Third, the methodology is singular. Most studies only employ basic bibliometric indicators and fail to integrate multi-dimensional network analysis and time evolution models.

Our study addresses these gaps by covering the complete period from 2016 to 2026, specifically capturing the critical impact of the COVID–19 pandemic, and creating a 4-dimensional analysis framework that covers “basic mechanisms-clinical symptoms-social factors-technology applications” to provide comprehensive coverage from molecular biology to public health strategies.

### 4.2. Main findings

Our analysis reveals major changes in depression research from 2016 to 2026: Global research output is highly concentrated (the United States produces 53.3% of articles, and top institutions contribute 43.5%). Research focus has shifted from traditional theories about brain chemicals to understanding interactions between the immune system, gut bacteria, and genetic factors. Treatment approaches have evolved to combine biological, psychological, social, and digital strategies. The COVID–19 pandemic (with emergency intensity >9.0) acted as a major turning point, fostering collaboration between biomedical science and data science, and creating new patterns for sharing knowledge through both traditional journals and open-access platforms. Research trends show “sudden changes followed by steady growth,” reflecting how external events and internal scientific progress work together to shape the field.

### 4.3. Limitations

The limitations of this study are mainly reflected in five aspects: a single data source (only based on Web of Science, ignoring other databases and non-English literature, there is a risk of cultural bias), limited timeliness of sample selection (highly cited literature standards lead to the omission of emerging research and overemphasis on historical influence), inherent flaws in methodology (quantitative indicators cannot evaluate qualitative dimensions such as research quality and methodological rigor), superficiality of collaboration network analysis (only reflects signature collaboration relationships and cannot assess collaboration quality and depth of resource integration), and interdisciplinary integration assessment challenges (collaboration frequency statistics are difficult to reflect the true degree of knowledge integration and innovation). These limitations point out the direction for improvement in future research, and it is recommended that follow-up work integrate multi-source databases, develop dynamic impact assessment models, and combine qualitative analysis methods to build a more comprehensive and in-depth analysis system of the depression research landscape.

## 5. Conclusions

Based on our analysis of 1000 highly cited depression articles from 2016 to 2026, this study reveals profound changes in depression research and the global scientific landscape. We found that research output is highly concentrated, with the United States leading production and China showing the highest citation impact per article, demonstrating strong research influence.

In terms of content, the field is moving from traditional theories about brain chemicals to understanding complex interactions between the nervous system, immune system, gut bacteria, and genetic factors. Treatment models have also advanced to integrate digital technology with biological, psychological, and social approaches.

Most importantly, the COVID–19 pandemic served as a major turning point, not only reshaping short-term research priorities but also driving deep collaboration between biomedical science and data science. Although our study has limitations due to using only the Web of Science database and focusing on highly cited articles, the comprehensive knowledge map we created provides valuable insights for understanding how depression research evolves, identifying new research frontiers, and making better decisions about future research investments.

## Acknowledgments

We would like to express our sincere gratitude to Tongji Medical College Union Wuhan Central Hospital, Huazhong University of Science and Technology, for providing research resources and platform support. Additionally, this work was supported by the following funding sources: in 2024, by the Higher Education Steering Committee of the Ministry of Education for the project titled “Evidence-Based Translational Medicine” (Project No. B_YXC2024-01-01_10), and in 2025, by the Health Commission of Hubei Province for the project named “AIGC-Driven Statistical Thinking to Create an Integrated Professional Postgraduate Training Model for Scientific Research-Clinical Transformation” (Project No. HBJG-250041).

During the preparation of this manuscript, the authors utilized Chat GPT and Deep Seek to assist with document drafting, language polishing, and initial ideation, while all core scientific work was independently conducted by the authors. The authors take full responsibility for all content of the manuscript and ensure its compliance with academic standards.

## Author contributions

**Conceptualization:** Jinlei Li.

**Formal analysis:** Fen Ai.

**Resources:** Yu Li.

**Software:** Bingxin Cheng.

**Validation:** Zhen Chen.



## References

[1] Kessler RC Bromet EJ. The epidemiology of depression across cultures. Annu Rev Public Health. 2013;34:119–38. 10.1146/annurev-publhealth-031912-114409 PMC4100461 23514317

[2] Moussavi S Chatterji S Verdes E Tandon A Patel V Ustun B. Depression, chronic diseases, and decrements in health: results from the World Health Surveys. Lancet. 2007;370:851–8. 10.1016/S0140-6736(07)61415-9 17826170

[3] Fusar‐Poli P Solmi M Brondino N. Transdiagnostic psychiatry: a systematic review. World Psychiatry. 2019;18:192–207. 10.1002/wps.20631 PMC6502428 31059629

[4] Shi S Gao Y Sun Y. The top-100 cited articles on biomarkers in the depression field: a bibliometric analysis. Psychol Health Med. 2021;26:533–42. 10.1080/13548506.2020.1752924 32290683

[5] Chen Y-J Zhu Q-Q Xie M-R. Microglia associated research in depression: an exploratory trends analysis. Front Aging Neurosci. 2026;18:1757943. 10.3389/fnagi.2026.1757943 PMC13012938 41890205

[6] Wu D Chen Y Izumoji G. Unveiling the relationship of the comorbidity between depression and type 2 diabetes mellitus: a macro analysis and micro interpretation. Front Med (Lausanne). 2026;13:1785271. 10.3389/fmed.2026.1785271 PMC13106598 42040575

[7] Xu D Wu J Lu Z. Bibliometric analysis of research hotspots and trends on the relationship between the gut microbiota and depression from 2020 to 2024. Front Microbiol. 2024;15:1479703. 10.3389/fmicb.2024.1479703 PMC11586207 39588103

[8] Wang X Qian Y He Q. Bibliometric analysis of the post-stroke depression (1985-2024). Brain Behav. 2026;16:e71345. 10.1002/brb3.71345 PMC13111916 42037146

[9] Sun Y Li J Wei H. Comparison of the intervention effects of different Tai Chi training intensities on fatigue-insomnia-depression triad in cancer patients: a bibliometric analysis review. Psycho-Oncol. 2026;20:4782.

[10] Hao Y Han Y Huang J. The application of electroencephalogram in depression research: bibliometric and technological application analysis from 2005 to 2025. Front Neurosci. 2025;19:1653693. 10.3389/fnins.2025.1653693 PMC12408689 40918982

[11] Shao D Shao L Kou Z. A data-driven analysis of artificial intelligence applications in depression research: 2020-2025. Asian J Psychiatr. 2026;120:104978. 10.1016/j.ajp.2026.104978 41996983

[12] Tian Y Wang S Li G Deng J Yang D Fang Y. Global research landscape and emerging trends in the application of artificial intelligence in depression: a bibliometric analysis. Digit Health. 2026;12:20552076261427866. 10.1177/20552076261427866 PMC12949316 41767874

[13] Lu E Zhang D Han M Wang S He L. The application of artificial intelligence in insomnia, anxiety, and depression: a bibliometric analysis. Digit Health. 2025;11:20552076251324456. 10.1177/20552076251324456 PMC11873874 40035038

[14] Wang H Tian X Wang X Wang Y. Evolution and emerging trends in depression research from 2004 to 2019: a literature visualization analysis. Front Psychiatry. 2021;12:705749. 10.3389/fpsyt.2021.705749 PMC8585938 34777037

[15] Zhou Q Pei J Poon J. Worldwide research trends on aristolochic acids (1957-2017): suggestions for researchers. PLoS One. 2019;14:e0216135. 10.1371/journal.pone.0216135 PMC6497264 31048858

[16] van Eck NJ Waltman L. Software survey: VOSviewer, a computer program for bibliometric mapping. Scientometrics. 2010;84:523–38. 10.1007/s11192-009-0146-3 PMC2883932 20585380

[17] Bibliometric Online Analysis Platform. https://bibliometric.com/. Accessed March 23, 2026.

[18] Aria M Cuccurullo C. bibliometrix : an R-tool for comprehensive science mapping analysis. J Informetr. 2017;11:959–75.

[19] K-Synth Srl. Bibliometrix: The Complete Science Mapping Platform. 2026. https://www.bibliometrix.org/home/. Accessed March 23, 2026.

[20] Synnestvedt MB Chen C Holmes JH. CiteSpace II: visualization and knowledge discovery in bibliographic databases. AMIA Annu Symp Proc. 2005;2005:724–8. PMC1560567 16779135

[21] Chen C. Searching for intellectual turning points: progressive knowledge domain visualization. Proc Natl Acad Sci U S A. 2004;101(Suppl 1):5303–10. 10.1073/pnas.0307513100 PMC387312 14724295

[22] Scimago Graphica. SCImago; 2026. https://www.graphica.app/. Accessed March 24, 2026.

[23] Center for Open Science. Open Science Framework. 2026. https://osf.io/. Accessed March 23, 2026.

[24] Brooks SK Webster RK Smith LE. The psychological impact of quarantine and how to reduce it: rapid review of the evidence. Lancet. 2020;395:912–20. 10.1016/S0140-6736(20)30460-8 PMC7158942 32112714

[25] Wang C Pan R Wan X. Immediate psychological responses and associated factors during the initial stage of the 2019 coronavirus disease (COVID-19) epidemic among the general population in China. Int J Environ Res Public Health. 2020;17:1729. 10.3390/ijerph17051729 PMC7084952 32155789

[26] Lai J Ma S Wang Y. factors associated with mental health outcomes among health care workers exposed to coronavirus disease 2019. JAMA Netw Open. 2020;3:e203976. 10.1001/jamanetworkopen.2020.3976 PMC7090843 32202646

[27] Xiong J Lipsitz O Nasri F. Impact of COVID-19 pandemic on mental health in the general population: a systematic review. J Affect Disord. 2020;277:55–64. 10.1016/j.jad.2020.08.001 PMC7413844 32799105

[28] Malhi GS Mann JJ. Depression. Lancet. 2018;392:2299–312. 10.1016/S0140-6736(18)31948-2 30396512

[29] Miller AH Raison CL. The role of inflammation in depression: from evolutionary imperative to modern treatment target. Nat Rev Immunol. 2016;16:22–34. 10.1038/nri.2015.5 PMC5542678 26711676

[30] GBD 2015 Disease and Injury Incidence and Prevalence Collaborators. Global, regional, and national incidence, prevalence, and years lived with disability for 310 diseases and injuries, 1990-2015: a systematic analysis for the Global Burden of Disease Study 2015. Lancet. 2016;388:1545–602. 10.1016/S0140-6736(16)31678-6 PMC5055577 27733282

[31] Ferrucci L Fabbri E. Inflammageing: chronic inflammation in ageing, cardiovascular disease, and frailty. Nat Rev Cardiol. 2018;15:505–22. 10.1038/s41569-018-0064-2 PMC6146930 30065258

[32] Solmi M Radua J Olivola M. Age at onset of mental disorders worldwide: large-scale meta-analysis of 192 epidemiological studies. Mol Psychiatry. 2022;27:281–95. 10.1038/s41380-021-01161-7 PMC8960395 34079068

[33] Ahorsu DK Lin CY Imani V Saffari M Griffiths MD Pakpour AH. The fear of COVID-19 scale: development and initial validation. Int J Ment Health Addict. 2022;20:1537–45. 10.1007/s11469-020-00270-8 PMC7100496 32226353

[34] Zhu X Hu J Deng S. Bibliometric and visual analysis of research on the links between the gut microbiota and depression from 1999 to 2019. Front Psychiatry. 2020;11:587670. 10.3389/fpsyt.2020.587670 PMC7819979 33488420

[35] Tran BX McIntyre RS Latkin CA. The current research landscape on the artificial intelligence application in the management of depressive disorders: a bibliometric analysis. Int J Environ Res Public Health. 2019;16:2150. 10.3390/ijerph16122150 PMC6617113 31216619

[36] Ai Y Xing Y Yan L. Atrial fibrillation and depression: a bibliometric analysis from 2001 to 2021. Front Cardiovasc Med. 2022;9:775329. 10.3389/fcvm.2022.775329 PMC8888833 35252380

[37] You Y Wang D Wang Y Li Z Ma X. A bird’s-eye view of exercise intervention in treating depression among teenagers in the last 20 years: a bibliometric study and visualization analysis. Front Psychiatry. 2021;12:661108. 10.3389/fpsyt.2021.661108 PMC8249759 34220574

[38] Wang X-Q Peng M-S Weng L-M Zheng Y-L Zhang Z-J Chen P-J. Bibliometric study of the comorbidity of pain and depression research. Neural Plast. 2019;2019:1657498. 10.1155/2019/1657498 PMC6854239 31772566

[39] Zyoud SH Shakhshir M Abushanab AS. Bibliometric mapping of the landscape and structure of nutrition and depression research: visualization analysis. J Health Popul Nutr. 2023;42:33. 10.1186/s41043-023-00378-2 PMC10105358 37061731

[40] Ying H Zhang X He T. A bibliometric analysis of research on heart failure comorbid with depression from 2002 to 2021. Heliyon. 2023;9:e13054. 10.1016/j.heliyon.2023.e13054 PMC9900509 36755587

[41] Zhang T Yang R Pan J Huang S. Parkinson’s disease related depression and anxiety: a 22-year bibliometric analysis (2000-2022). Neuropsychiatr Dis Treat. 2023;19:1477–89. 10.2147/NDT.S403002 PMC10317541 37404573

[42] Al-Jabi SW. Current global research landscape on COVID-19 and depressive disorders: bibliometric and visualization analysis. World J Psychiatry. 2021;11:253–64. 10.5498/wjp.v11.i6.253 PMC8209539 34168972

